# Factors associated with dengue prevention behaviour in Lowokwaru, Malang, Indonesia: a cross-sectional study

**DOI:** 10.1186/s12889-018-5553-z

**Published:** 2018-05-11

**Authors:** Alidha Nur Rakhmani, Yanin Limpanont, Jaranit Kaewkungwal, Kamolnetr Okanurak

**Affiliations:** 1 Department of Social and Environmental Medicine, Faculty of Tropical Medicine, Mahidol University, Bangkok, Thailand; 2 Faculty of Medicine, Brawijaya University Malang, Malang, Indonesia; 3 Department of Tropical Hygiene, Faculty of Tropical Medicine, Mahidol University, Bangkok, Thailand

**Keywords:** Dengue, Prevention, Behaviour, Urban, Malang, Indonesia

## Abstract

**Background:**

Dengue prevention is important for controlling the spread of dengue infection. Transmission of dengue can be prevented by controlling mosquito breeding sites. Indonesia has dengue a prevention program to minimize mosquito breeding sites known as 3 M Plus. This study aimed to investigate factors associated with dengue prevention behaviour among respondents in the Lowokwaru subdistrict, an urban area in Malang, Indonesia.

**Methods:**

This cross-sectional study used a semi-structured questionnaire that was conducted by face-to-face interview.

**Results:**

Older respondents (> 60 years and 41–60 years) showed better dengue prevention behaviour than younger respondents (21–40 years and < 21 years) (*p* value = 0.01). Proportionally more male respondents showed poor dengue prevention behaviour compared with female respondents (*p* value = 0.007). Respondents who lived in Malang for long durations showed better dengue prevention behaviour compared with those who lived there for a shorter period (*p* value = 0.016). Those with more family members in their households practiced better dengue prevention behaviour compared with those with fewer family members (*p* value = 0.004). Perception was associated with dengue prevention behaviour. Respondents who had higher perceived susceptibility showed better dengue prevention behaviour compared with those who had moderate perceptions (*p* value = 0.000).

**Conclusions:**

Age, gender, duration of stay in Malang, number of family members, and perception of dengue susceptibility were associated with dengue prevention behaviour.

## Background

Dengue is regarded by the World Health Organization as “one of the most important arboviral infections in the world” [1]. The burden of dengue has grown dramatically, particularly in Southeast Asia and the Western Pacific [2]. Several factors influenced this resurgence, including: 1) population growth in urban areas resulting in substandard housing and inadequate water and waste management systems [3]; 2) lack of education, information and communication concerning vectors of dengue virus [4]; 3) increased air travel, which has allowed for the rapid movement of infected travellers between population centres of the tropics and resulted in an exchange of dengue viruses [5]; and 4) ineffective mosquito control measures for reducing the mosquito population [6, 7].

The first dengue cases were reported in Jakarta and Surabaya in 1964. In total, 58 patients were diagnosed with dengue in Surabaya, 24 of whom subsequently died. Thereafter, dengue spread to all provinces of Indonesia [8, 9]. By 2010, Indonesia ranked the highest for dengue cases among Southeast Asian countries [10]. In 2014, the Ministry of Health reported that the regions with the highest incidence of dengue cases were West, Central and East Java [11].

In 1992, the Ministry of Health of Indonesia began implementing various strategies against dengue, including surveillance systems, case management, vector control and programs aimed at changing people’s behaviour. The aims of vector control and behavioural change were combined in a surveillance program known as 3 M Plus, which required communities to be responsible for periodically finding and eradicating potential and existing mosquito nests in their respective vicinities. The three “M’s” were: *menutup* (meaning covering water containers), *menguras* (cleaning water containers), and *mengubur/membuang* (burying/throwing discarded items). Meanwhile, the “Plus” indicates activities aimed at reducing mosquito breeding places, such as using chemicals to kill larvae or fogging, and activities to protect people from mosquito bites, such as using repellent, mosquito coils, insecticide or long sleeves and trousers [8, 12].

Malang is the second largest city in East Java Province, Indonesia. Malang has been an endemic area of dengue since the first time dengue occurred in Indonesia [8, 9]. The number of cases of dengue in Malang continues to grow. The worst outbreak of dengue in Indonesia occurred in 2010 and urban areas like Malang were especially hard hit. There were 658 dengue cases from January to May 2010 in Malang, with 243 cases reported in February alone. Moreover, dengue-related mortality cases have increased every year; one patient died in 2014, whereas three patients died in 2015 [13–15].

Previous studies have revealed that many factors influence dengue prevention practices, including knowledge and perception. Participants with higher knowledge of dengue were reported to adopt dengue prevention techniques more frequently compared with those with low knowledge [16–21]. In addition, studies found that individuals with high perceived susceptibility to dengue adopted more dengue prevention measures compared with those with low perceived susceptibility [17]. A study in Malaysia showed the prevalence of dengue fever was higher in participants who engaged in high-risk behaviours compared with those who exhibited low-risk behaviours [21]. However, several studies revealed that good knowledge about dengue fever did not correlate with good prevention behaviour [22–24].

Regarding socioeconomic factors, such as income, there was an association between family income and dengue prevention behaviour [25]. Another study revealed an association between number of family members and dengue prevention behaviour [26]. Therefore, this study aimed to investigate factors associated with dengue prevention behaviour among respondents living in high-risk urban areas in Malang. This study would be useful for local health providers in planning appropriate interventions to increase community participation in dengue prevention programs, particularly in urban areas in Malang.

## Methods

### Study design and sample

Sample size was calculated by using the formula ${\mathrm{n}}_0=\kern0.5em \frac{{\mathrm{Z}}^2\mathrm{pq}}{{\mathrm{e}}^2}$ [27].

Where n_0_ is the sample size, z^2^ was standard value normal distribution at 95% confidence level (1.96), p (=42.6) was percentage of dengue prevention behaviour from previous study [28], e^2^ was acceptable maximum error (7%). The minimum sample size was 191 and after adding 20% to anticipate for the non-response rate, data were collected from 220 respondents.

This was a cross-sectional study conducted among respondents living in Malang City of East Java during May–June 2016. Malang city has divided area into urban (sub-district) and rural (regency). There are five sub-districts in Malang: Lowokwaru, Klojen, Blimbing, Kedungkandang and Sukun. Lowokwaru sub-district with the highest number of dengue cases was selected as studied site. In 2015, Lowokwaru sub-district had 50,574 household with the total population of 166,837 [29]. This sub-district consists of 12 villages. This study was conducted in Lowokwaru village with the highest prevalence of dengue in 2015. Lowokwaru village has 104 clusters [30]. This study randomly selected 15 clusters from Lowokwaru village. From each cluster, 14–15 respondents were randomly recruited into the study. This study enrolled registered residents aged 18 years and above who had lived more than 6 months in the Lowokwaru village and who were willing to participate in this study. Residents who were not at home during data collection were excluded from this study.

### Data collection and analysis

Data collection to explore knowledge, perceptions, and dengue prevention behaviour used a semi-structured questionnaire conducted through face-to-face interviews. The questionnaire was developed, pre-tested and modified according to the pre-tested results. Reliability was computed using Cronbach’s alpha coefficient and resulted in alpha score 0.736 [31]. Before interviewing, all respondents were informed of the study details and that the information they gave would be kept confidential and they could stop answering questions at any time. The maximum score for the knowledge portion was 14 and respondents would be considered as needing improvement if they scored 0–8 (< 60%), having moderate knowledge if they scored 9–11 (60–80%), and having good knowledge with a score of 12–14. The perception portion consisted of 19 statements. Each positive statement was given a score of 5 for “strongly agree”, 4 for “agree”, 3 for “neutral”, 2 for “disagree”, and 1 for “strongly disagree”. Conversely, negative statements were given a score of 5 for “strongly disagree”, 4 for “disagree”, 3 for “neutral”, 2 for “agree”, and 1 for “strongly agree”. The maximum score for the perception portion was 95 and respondents scoring 0–31 were considered to have a negative perception, those scoring 32–63 were considered to have a moderate perception, and a score of 64–95 indicated a positive perception if the score range. The maximum score for dengue prevention behaviour was 18, and participant were considered as needing improvement with a score of 0–10 (< 60%), showing moderate prevention behaviours with a score of 11–14 (60–80%), and good prevention behaviour with a score of 15–18. SPSS, Version 18 was used for statistical analysis, and descriptive data were presented as frequencies and percentages.

SPSS was used for analyses and chi-square was used to explore the association between independent variables and dengue prevention behaviour. Factors influencing dengue prevention behaviour were analysed using binary logistic regression model, backward.

## Results

Approximately 72% of respondents were female; 67.7% were aged 31–60 years at the time of the study, and 80% reported having lived in Malang for more than 20 years. Almost one-third of the sample completed senior high school or higher education; 57.3% were unemployed; and 67% of those employed had an income of 1–3 million rupiah per month. Approximately 90% of respondents reported having three or more family members in their household (Table 1).

**Table 1:** Respondent characteristics

	Number	Percent
Gender		
Male	60	27.3
Female	160	72.7
Age		
18–30	40	18.2
31–40	48	21.8
41–50	49	22.3
51–60	52	23.6
> 60	31	14.1
Duration of stay in Malang		
< 21 years	44	20.0
21–30 years	32	14.5
31–40 years	47	21.4
41–50 years	41	18.6
51–60 years	35	15.9
> 60 years	21	9.6
Education level		
No education	2	0.9
Elementary school	23	10.5
Junior high school	33	15.0
Senior high school	117	53.2
College and higher	45	20.5
Occupation		
Unemployed	126	57.3
Government	6	2.7
Private (owner)	31	14.1
Private (worker)	57	25.9
Income/month (million rupiah^*^)		
< 1	18	19.1
1–3	63	67.0
> 3	13	13.8
Number of family members in the household (person)		
1–2	22	10.1
3–4	99	45.4
> 4	97	44.5

^*^Million rupiah = 100 US$

Approximately 88% of respondents reported having received information about dengue, and about 25% had experienced of dengue (Table 2).

**Table 2:** Information and experience of dengue

Issue	Number	Percent
Received dengue information		
Yes	193	87.7
No	27	12.3
Family experience of dengue		
Yes	56	25.5
No	164	74.5

It was found that 43.6% of respondents had moderate knowledge and 36.4% had good knowledge regarding dengue. More than 82% of respondents had a positive perception of dengue. Most had a moderate level of prevention behaviour. Only 3.2% of respondents had a good level of prevention behaviour, and 35.8% needed to improve their prevention behaviour (Table 3).

**Table 3:** Level of knowledge, perception, and prevention behaviour regarding dengue

Issue	Number	Percent
Knowledge level		
Need Improvement	44	20
Moderate	96	43.6
Good	80	36.4
Perception level		
Moderate	39	17.7
Positive	181	82.3
Prevention behaviour level		
Need improvement	78	35.8
Moderate	133	61.0
Good	7	3.2

### Dengue prevention behaviour and associated factors

Older respondents (> 60 years and 41–60 years) showed better dengue prevention behaviour than younger respondents (21–40 years and < 21 years) (*p* value = 0.01). A larger portion of male respondents needed to improve their dengue prevention behaviour compared with female respondents (*p* value = 0.007). Those who stayed longer in Malang reported better dengue prevention behaviour than those who lived in Malang for shorter durations (*p* value = 0.016). Those with more family members showed better dengue prevention behaviour compared with those with less family members (*p* value = 0.004). It was found that dengue prevention behaviour is associated with perceptions. Respondents who had a positive perception showed better dengue prevention behaviour compared with those who had a moderate perception (*p* value = 0.000). Those who positively perceived benefit toward dengue prevention practiced prevention behaviour more than those who had moderate level (p value = 0.005). It was also found that respondents who had positive level of barrier toward dengue prevention had better prevention behaviour than those who had moderate level (p value = 0.000) (Table 4).

**Table 4:** Association between independent factors and dengue prevention behaviour

Independent variables	Dengue prevention behaviour		P value
	Need improvement	Moderate and Good^*^	
	N (%)	N (%)	
Gender			
Male	30 (50%)	30 (50%)	0.007
Female	48 (30.4%)	110 (69.6%)	
Age			
< 21 years	4 (80.0%)	1 (20.0%)	0.010^**^
21–40 years	37 (44.6%)	46 (55.4%)	
41–60 years	31 (31.0%)	69 (69.0%)	
> 60 years	6 (20.0%)	24 (80.0%)	
Duration of stay in Malang (year)			
1–20	23 (52.3%)	21 (47.7%)	0.016
21–40	31 (39.2%)	48 (60.8%)	
40–60	20 (26.7%)	55 (73.3%)	
> 60	4 (20.0%)	16 (80.0%)	
Number of family member (year)			
1–2	15 (68.2%)	7 (31.8%)	0.004
3–4	34 (34.35)	65 (65.7%)	
> 4	29 (30.5%)	66 (69.5%)	
Perception level			
Moderate	24 (61.5%)	15 (38.5%)	0.000
Positive	54 (30.2%)	125 (69.8%)	
Perceive benefit level toward prevention behaviour			
Moderate	27 (51.9%)	25 (48.1%)	0.005
Positive	51 (30.7%)	115 (69.3%)	
Perceive barrier level toward prevention behaviours			
Moderate	50 (51%)	48 (49%)	0.000
Positive	28 (23.3%)	92 (76.7%)	

^*^Moderate combined with good dengue prevention behaviour

^**^Fisher’s exact test

Female respondents showed better (2.18 times) dengue prevention behaviour compared with male respondents. Respondents who had 3–5 and > 5 family members in their respective households showed better dengue prevention behaviour (7.08 times and 6.71 times, respectively) compared with those with 1–2 family members. Respondents who lived in Malang longer showed better dengue prevention behaviour. Respondents who lived in Malang for 21–40 years showed prevention behaviour 10.57 times better than those who lived in Malang for 1–20 years. It was also found that respondents who had a positive perception performed better prevention behaviour (3.74 times) compared with those who had a moderate perception. Respondents who had positive level of benefit and barrier toward dengue prevention showed better prevention than behaviour those who had moderate level (3.094 times and 2.285 times, respectively) (Table 5).

**Table 5:** Binary logistic regression analysis of factors influencing dengue prevention behaviour^*^

Variable	OR	95% CI	*P* value
Gender			
Female	2.18	1.104–4.296	0.025
(Reference: male)	1.00		
Number of family member (person)			
> 5	6.71	2.181–20.666	0.02
3–5	7.08	2.281–21.997	0.01
(Reference: 1–2)	1.00		0.01
Duration of stay (year)			
> 60	1.40	0.624–3.160	0.41
41–60	2.73	1.163–6.403	0.21
21–40	10.57	2.268–49.257	0.03
(Reference: 1–20)	1.00		0.07
Perception level			
Positive	3.74	0.139–0.624	0.01
(Reference: moderate)	1.00		
Perceive benefit level toward prevention behaviour			
Positive	3.094	1.362–7.028	0.007
(Reference: moderate)			
Perceive barrier level toward prevention behaviours			
Positive	2.285	1.160–4.499	0.017
(Reference: moderate)			

^*^Moderate combined with good dengue prevention behaviour

## Discussion

Previous studies did not find an association between gender and dengue prevention behaviour [21, 26, 28–32]. However, this study showed a significant association between respondent gender and dengue prevention behaviour. Approximately 50% of male respondents performed at the “need improvement” level of prevention behaviour, compared with female respondents at 30.4%. Indeed, most female respondents showed good prevention behaviour. This might be because in Indonesia, females generally have the social role of caring for the family and household [33]. Moreover, there is a working women’s group (PKK) that engages in social community activities, such as delivering dengue information [12].

It was found that dengue prevention behaviour was associated with the number of family members in a household. This result is consistent with those of other studies [26, 32]. A previous study found that respondents with a 3–5-person household and those with a > 5-person household showed better prevention behaviour compared with those with a 1–2-person household [32]. Similar result was reported for Aceh, Indonesia. It was found that those with more than five family members exhibited better behaviour compared with those with 1–5 members [26]. This might be because more family members could contribute to housekeeping tasks, including dengue prevention activities. It could also be that families with more members were able to acquire and share dengue information from more sources [26].

Studies in Thailand reported no association between duration of stay and dengue prevention behaviour [32, 34]. However, in the present study, duration of stay in Malang was associated with dengue prevention behaviour. Respondents who stayed longer practiced good behaviours more than those who resided in Malang for shorter periods of time. Fewer respondents living in Malang for > 60 years were categorized as “need improvement” for dengue prevention behaviour compared with those who had stayed in Malang for < 60 years. This might be because those who stayed > 60 years had more experience of dengue. Another study also revealed that duration of stay is associated with dengue prevention behaviour [28].

This study revealed that perceptions are associated with dengue prevention behaviour. Respondents with a positive perception engaged in dengue prevention behaviour more than those with a moderate perception. Furthermore, the study found that respondents who had positive level toward benefit and barrier of prevention had practiced prevention behaviour more than those with moderate levels. A study in Malaysia also found respondents with higher perceived susceptibility showed better dengue prevention behaviour [9].

Results concerning prevention behaviours were limited only on the interview data, the study did not follow up or observe respondents’ prevention actions. Another limitation was that in Muslim culture a third person presented during the interview. This might cause information bias.

This study showed that positive perception is the key component affected prevention behaviours, particularly perception toward benefit and barrier of prevention behaviours. It is, therefore, increase people’s perception regarding dengue is the main component in changing people’s prevention behaviours. Health providers have to find an appropriate and effective strategy to create people’s positive perception concerning dengue infection.

## Conclusions

Age, gender, duration of stay in Malang, and number of family members were associated with dengue prevention behaviour. Those who had a positive perception showed better dengue prevention behaviour compare with those with a moderate perception.
